# Bilateral Common Carotid Artery Stenosis in Mice: A Model of Chronic Cerebral Hypoperfusion-Induced Vascular Cognitive Impairment

**DOI:** 10.21769/BioProtoc.5022

**Published:** 2024-07-05

**Authors:** Masashi Kakae, Ayaka Kawashita, Haruya Onogi, Takayuki Nakagawa, Hisashi Shirakawa

**Affiliations:** 1 Department of Molecular Pharmacology, Graduate School of Pharmaceutical Sciences, Kyoto University, Kyoto, Japan; 2 Department of Clinical Pharmacology and Pharmacotherapy, School of Pharmaceutical Sciences, Wakayama Medical University, Wakayama, Japan

**Keywords:** Bilateral common carotid artery stenosis (BCAS), Vascular cognitive impairment (VCI), Chronic cerebral hypoperfusion (CCH), White matter injury, Microcoil, Vagus nerve

## Abstract

Vascular cognitive impairment (VCI) is a syndrome defined as cognitive decline caused by vascular disease and is associated with various types of dementia. Chronic cerebral hypoperfusion (CCH) is one of the major contributors to VCI. Among the various rodent models used to study CCH-induced VCI, we have found the mouse bilateral common carotid artery stenosis (BCAS) model to be highly suitable. Here, we introduce the BCAS model of C57BL/6J mice generated using microcoils with an internal diameter of 0.18 mm. To produce the mouse BCAS model, the bilateral common carotid arteries are isolated from the adhering tissues and vagus nerves and twined around the microcoils. This model shows cognitive impairment and white matter lesions preceding neuronal dysfunction around postoperative day 28, which is similar to the human clinical picture. Overall, the mouse BCAS model will continue to be useful in studying CCH-induced VCI.

Key features

• This mouse BCAS model requires approximately 4 weeks to show phenotypes such as cognitive impairment and white matter injury.

## Graphical overview


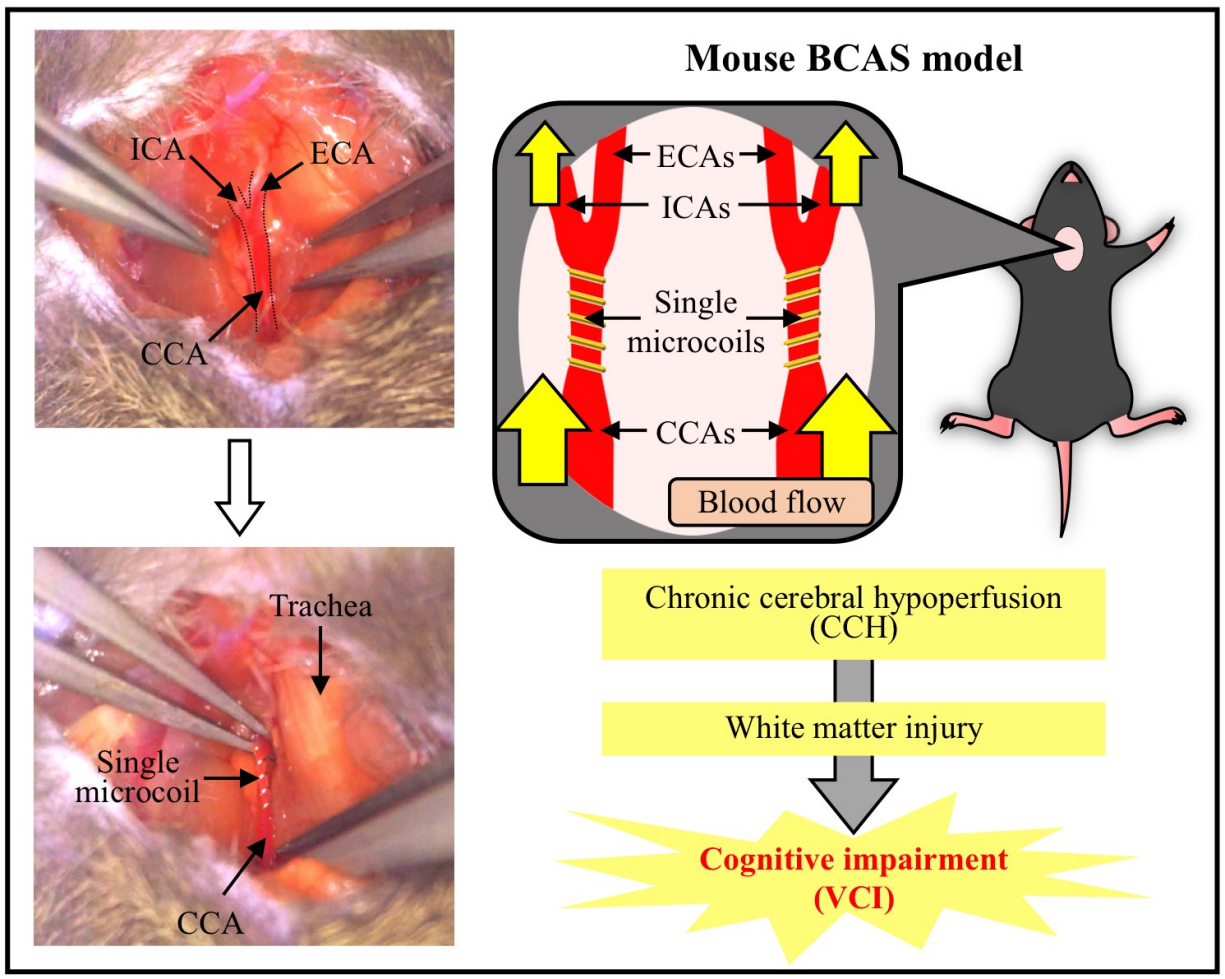


**Overview of the mouse BCAS model.** The BCAS-operated mouse develops CCH and white matter injury, leading to cognitive impairment. This BCAS model is considered to be a suitable and useful CCH-induced VCI model. BCAS, bilateral common carotid artery stenosis; CCA, common carotid artery; CCH, chronic cerebral hypoperfusion; ECA, external carotid artery; ICA, internal carotid artery; VCI, vascular cognitive impairment.

## Background

Vascular cognitive impairment (VCI) refers to cognitive alterations related to vascular disease and is associated with various types of dementia [1]. Chronic cerebral hypoperfusion (CCH)-associated small vessel disease is one of the major contributors to VCI [2,3]. CCH is elicited by aging and various lifestyle diseases, such as metabolic syndromes, atherosclerosis, hypertension, obesity [4], and hypotension [5]. It has also been suggested that CCH induces white matter lesions, which are key characteristics of VCI. In addition, many patients with various types of dementia including VCI have white matter lesions [6]. To understand the precise pathological mechanism underlying CCH-induced VCI including white matter lesions, multiple rodent models have been used in many studies.

Some widely used rodent models of CCH-induced VCI are the rat bilateral common carotid artery occlusion (BCCAO), mouse unilateral common carotid artery occlusion (UCCAO), rat and mouse 2-vessel gradual occlusion (2-VGO), mouse asymmetric common carotid artery surgery (ACAS), and mouse bilateral common carotid artery stenosis (BCAS) models. However, rodent models have several limitations. First, the visual pathway is damaged and behavioral tests to assess cognitive function are affected in the rat BCCAO and mouse UCCAO models [7,8]. Second, the device used in the rat and mouse 2-VGO models and mouse ACAS model is expensive [9]. The mouse BCAS model was reported as a CCH model in 2004 by Shibata et al. [10] and is a more suitable and useful tool for research of CCH-induced VCI. This model shows cognitive impairment and a decrease in myelin sheaths without severe damage to the visual pathway [9]. In addition, BCAS-induced cognitive impairment and white matter lesions precede neuronal death [11,12]. Therefore, the mouse BCAS model reproduces the clinical picture in human VCI patients [13]. However, surgery is harder to perform in this model than in the rat BCCAO and mouse UCCAO models, and expertise is needed to create a stable mouse model. Therefore, we present a detailed and robust method that can stably create the mouse BCAS model used in our previous studies [11,12,14] for further development of VCI research.

## Materials and reagents

1. Mice: Male C57BL/6J, 8–12 weeks old, 20–30 g
2. Isoflurane (Viatris, catalog number: 901036504)

## Equipment

1. Animal anesthetizer (Biomachinery, catalog number: TK-7)
2. Surgical microscope (Carl Zeiss, catalog number: OPMI11/S21)
3. Surgical equipment and materials (Figure 1)
  - Curved suture needle (Natsume Seisakusho, catalog number: C-24-500-1)
  - 5-0, white braided silk sutures (Akiyama Medical, catalog number: EWB0514)
  - Needle holder (SHIN-EI, catalog number: MNH-1)
  - Micro spring scissors (Natsume Seisakusyo, catalog number: MB-56)
  - Two forceps, straight sharp (Fine Science Tools, catalog number: 11293-00)
  - Graefe forceps (Fine Science Tools, catalog number: 11051-10)
  - Microcoils with a wire diameter of 0.08 mm, an internal diameter of 0.18 mm, a pitch of 0.50 mm, and a total length of 2.5 mm (Name abbreviation: microcoil 0.08 × 0.18 × 0.50 × 2.5), which are custom-made (Sawane Spring or Komatsu Spring). To obtain microcoils, contact the manufacturers in English via e-mail or website form as follows:
    Sawane Spring Co., Ltd.
    Website: https://www.sawane-spring.com
    Contact form: https://www.sawane-spring.com/cgi-bin/contact/form.cgi
    E-mail address: soudan@sawane.co.jp
    Komatsu Spring Industrial Co., Ltd.
    Website: https://www.komatsubane.com/english/
    Contact form: https://www.komatsubane.com/english/contact_us/
  - Handmade wire hook [kite string-tied bent 27 G needle with the tips cut off (TERUMO, catalog number: NN-2719)]
  - Paper towel (NIPPON PAPER CRECIA, catalog number: 61001)
    **Figure 1.**
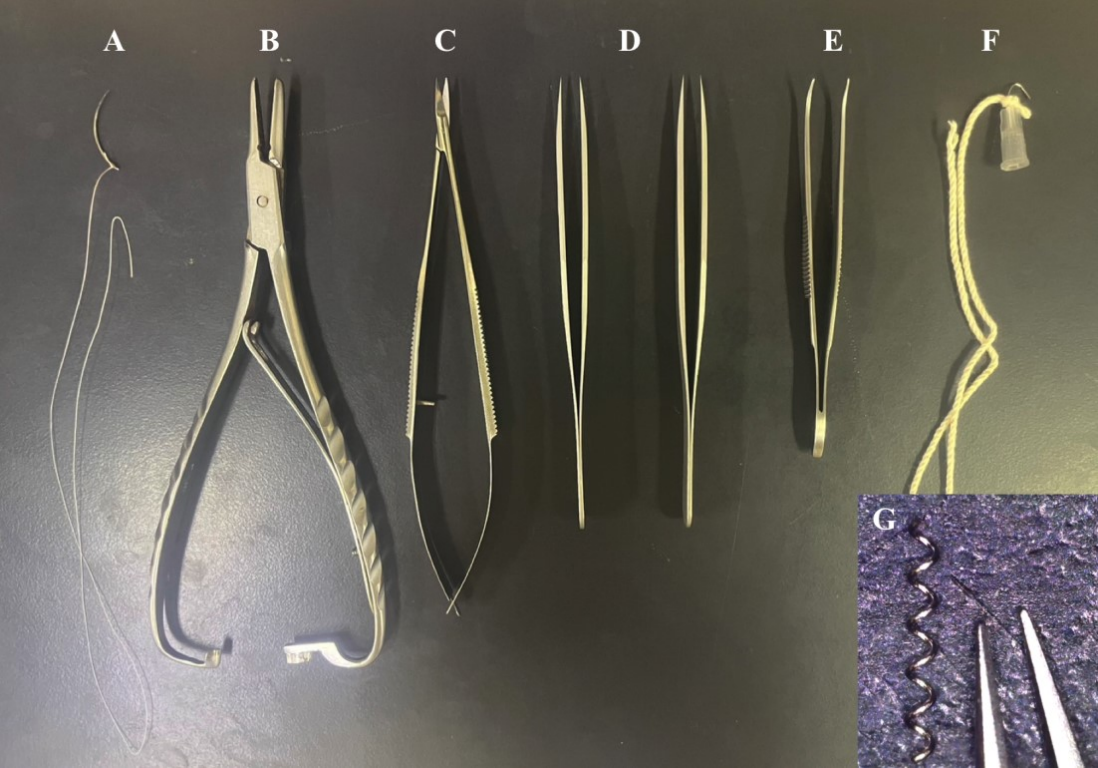
Surgical equipment and materials. A. Curved suture needle and silk suture. B. Needle holder. C. Micro spring scissors. D. Two straight sharp forceps. E. Curved forceps. F. Handmade wire hook. G. Microcoil with an internal diameter of 0.18 mm.

## Procedure

- **Anesthesia (presumably, any would be possible)**
  1. Anesthetize the mice with 3% isoflurane in 30% O_2_ and 70% N_2_O for ~3 min in the incubation box.
  2. Maintain anesthesia throughout surgery with 1.5% isoflurane in 30% O_2_ and 70% N_2_O using a face mask.
- **Isolation of the common carotid artery (CCA)**
  1. Place an anesthetized mouse in the supine position with the four limbs spread out.
  2. Make a ~1–1.5 cm ventral cervical skin incision in the midline (Figure 2A).
  3. Separate the submandibular glands laterally to make the trachea visible (Figure 2B).
  4. Move the sternocleidomastoid muscle laterally to make the CCA sheath visible by a handmade wire hook with a weight to open the surgical area (Figure 2C).
  5. Carefully isolate the CCA from the adhering tissues and vagus nerve using forceps. Be careful not to damage the CCA and vagus nerve (Figure 2D, Video 1).
*Note: The CCA and vagus nerve are wrapped in a transparent thin membrane, and the vagus nerve is often seen at a deep depth (dorsal) and on the lateral side of its sheath (Figure 2E).*
    **Figure 2.**
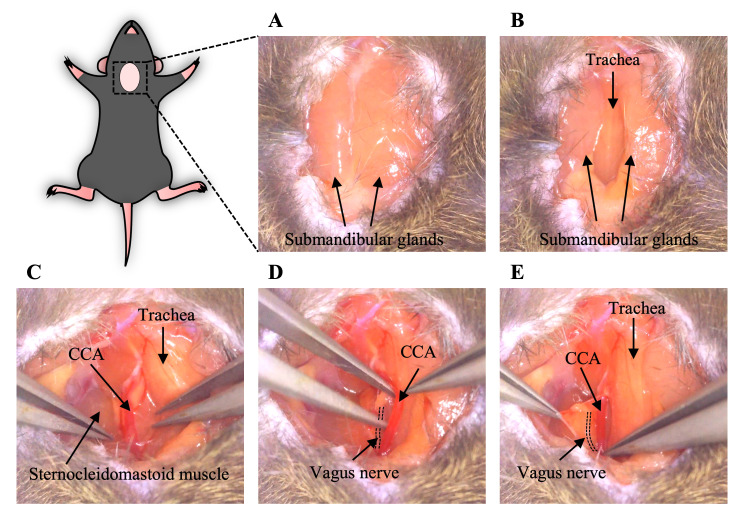
Generation of a ventral cervical skin incision and isolation of the CCA (section B). A. A cervical skin incision was made, and the submandibular glands were visualized. B. The submandibular glands were separated, and the trachea was exposed. C. The surgical area was opened by moving the sternocleidomastoid muscle laterally and the CCA was visible. D. The CCA was isolated from the adhering tissues and vagus nerve with forceps. E. Before isolation of the CCA, the vagus nerve was wrapped in a transparent membrane with the CCA. The dotted line shows the vagus nerve. CCA, common carotid artery.
- **Putting microcoils on the CCAs**
  1. Gently lift the isolated CCA and put a microcoil under it vertically. Put the CCA in the center of the microcoil (Figure 3A, Video 1).
  2. Grab one side of the microcoil and twine the CCA around the other side of the microcoil (Figure 3B, Video 1).
  3. Twine the CCA around the other side of the microcoil in the same way as described in step C2 (Figure 3C, Video 1).
  4. Remove the wire hook and place the sternocleidomastoid muscle and submandibular glands back.
  5. Repeat the procedure described in steps C3 and C4 with the other CCA. The other CCA is on the other side of the trachea at the symmetrical position. As described in step B4, the other CCA sheath is also visible after moving the other sternocleidomastoid muscle laterally.
    **Figure 3.**
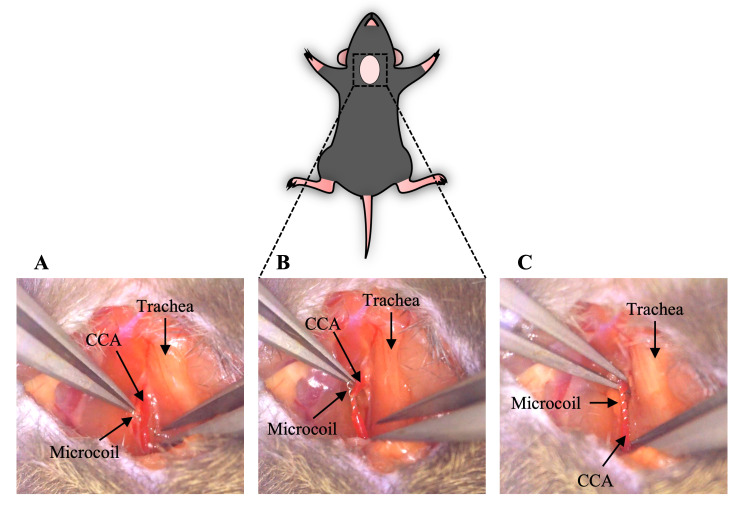
Placement of a microcoil on the CCA and completion of stenosis (section C). A. A microcoil was put under the isolated CCA. B. The CCA was twined around half of the microcoil. C. The CCA was twined around the other side of the microcoil, and stenosis was completed. CCA, common carotid artery.
    **Video 1.**
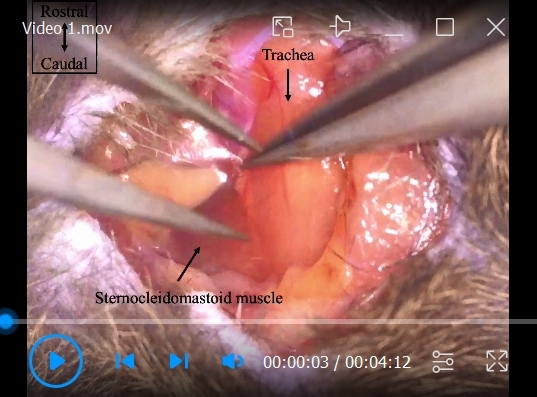
Surgical procedure for the isolation of the CCA and stenosis by a microcoil (steps B5–C3)
- **Completion of surgery**
  1. Suture the surgical cervical skin incision with a 5-0 silk suture in three interrupted sutures.
  2. Put the mouse back into a clean cage and house the mice at a constant ambient temperature of 22 ± 2 °C under a 12/12 h light/dark cycle. Allow them food and water ad libitum.
- **Procedure of the sham operation**
  1. Conduct the procedure described in steps A1–B5.
  2. Place the sternocleidomastoid muscle and submandibular glands back in the same way as described in step C4 without putting a microcoil on the CCA and repeat for the other CCA.
  3. Suture the surgical incision and put the mouse back into the cage as described in steps D1 and D2.

## Data analysis

We measured regional cerebral blood flow (rCBF) at 60 min after surgery with laser Doppler flowmetry [12]. BCAS surgery successfully reduced rCBF to ~65% of the baseline, which is similar to the data reported by Shibata et al. [10]. In our previous studies, this mouse BCAS model generated using wildtype mice (8–12 weeks old, 20–30 g) showed a decrease in the myelin density by myelin staining and cognitive impairment in a novel object recognition test on postoperative day 28, whereas there were no changes in the number of neuronal cells detected by immunostaining or spatial memory in a novel location recognition test [11,12,14].

## Validation of protocol

This protocol or parts of it has been used and validated in the following research articles:

- Shibata et al. [10]. White Matter Lesions and Glial Activation in a Novel Mouse Model of Chronic Cerebral Hypoperfusion. *Stroke* (Figures 1A–C, 2B, and Table 1).
- Miyanohara et al. [11]. TRPM2 Channel Aggravates CNS Inflammation and Cognitive Impairment via Activation of Microglia in Chronic Cerebral Hypoperfusion. *J Neurosci* (Figures 1F and 2A, B, D, E).
- Kakae et al. [12]. The astrocytic TRPA1 channel mediates an intrinsic protective response to vascular cognitive impairment via LIF production. *Sci Adv* (Figure 1P–R and supplementary figure 1D).

## General notes and troubleshooting

1. Do not damage CCAs and the vagus nerves. In particular, carefully conduct the procedures during isolation and twining of CCAs.
2. Fully twine CCAs on microcoils. If CCAs are positioned off the end of microcoils, re-twine them or rotate microcoils.
3. Conduct the procedures as quickly as possible. If possible, complete this surgery within ~10–20 min and take at most 30 min.
4. Put microcoils on the lower branch of the carotid artery, not the internal carotid artery or external carotid artery.
